# Reading Aloud

**Published:** 1876-02

**Authors:** 


### READING ALOUD.

THIS is an accomplishment possessed by so few that a good reader is almost as rare as a man of common-sense. It is greatly to be regretted that so little attention is paid to a branch of education so agreeable, so important, and so useful. Months of time and multitudes of dollars are expended on studies which could be profitably dispensed with altogether, while the cultivation of the ability to read aloud gracefully is very sadly neglected—in fact, is not considered as by any means an important acquisition. A beautiful singer delights a whole assembly, a beautiful reader not only delights but instructs. A fool may sing divinely. But a good reader must possess mind. Let the parents then, whose daughters have no taste for music, no ear for song, but who have hearts and intellects worthy of any man, give them a chance of showing what they are made of, a chance of making their way in the world, of cultivating the habit of reading aloud with care, with grace, with understanding, and thus put it in their power of bearing their part in the entertainment of any company into which they may be thrown.

But it is to the physical benefits to be derived from reading aloud, to which the attention is more particularly called. It is one of those exercises which combines mental and muscular effort, and hence has a double advantage. It is an accomplishment which may be cultivated alone, perhaps better alone than under a teacher, for then, a naturalness of intonation will be acquired from instinct rather than from art; the most that is required being that the person practising should make an effort to command the mind of the author, the sense of the subject.

To read aloud well, a person should not only understand the subject, but should hear his own voice and feel within him that every syllable was distinctly enunciated, while there is an instinct presiding which modulates the voice to the number or distance of the hearers. Every public speaker ought to be able to tell whether he is distinctly heard by the farthest auditor in the room; if he is not, it is from a want of proper judgment and observation.

Reading aloud helps to develop the lungs just as singing does if properly performed. The effect is to induce the drawing of long breaths every once in a while, oftener and deeper than if reading without enunciating. These deep inhalations never fail to develop the capacity of the lungs in direct proportion to their practice.

Common consumption begins uniformly with imperfect, insufficient breathing; it is the characteristic of the disease that the breath becomes shorter and shorter, through weary months, down to the close of life, and whatever counteracts that short breathing, whatever promotes deeper inspirations, is curative to that extent, inevitably and under all circumstances. Let any person make the experiment by reading this page aloud, and in less than three minutes, the instinct of a long breath will show itself. This reading aloud develops a weak voice, and makes it sonorous. It has great efficiency also in making the tones clear and distinct, freeing them from that annoying hoarseness, which the unaccustomed reader exhibits before he has gone over half a page, when he has to stop and hem and clear away, to the confusion of himself, as much as that of the subject.

This loud reading when properly done, has a great agency in educing vocal power, on the same principle that all muscles are strengthened by exercise, those of the voice-making organs being no exception to the general rule. Hence in many cases absolute silence diminishes the vocal power just as the protracted non-use of the arm of the Hindoo devotee, at length paralyzes it forever. The general plan in appropriate cases is to read aloud in a conversational tone thrice a day, for a minute or two, or three at a time, increasing a minute every other day, until half an hour is thus spent at a time, thrice a day, which is to be continued until the desired object is accomplished. Managed thus, there is safety and efficiency as a uniform result.

As a means then of health, of averting consumption, of being useful and entertaining in any company; as a means of showing the quality of the mind, let reading aloud be considered an accomplishment more indispensable than that of smattering French, of lisping Italian, of growling Dutch, or dancing cotillions, gallopades, polkas, and quadrilles.

